# Supplementary material for: Optimal protamine dosing after cardiopulmonary bypass: The PRODOSE adaptive randomised controlled trial
Source: PLoS Med. 2021 Jun 7;18(6):e1003658. doi: 10.1371/journal.pmed.1003658 (PMC8216535; doi:10.1371/journal.pmed.1003658)
Supplement: S1 Appendix — ICU, intensive care unit. (DOCX) [file pmed.1003658.s001.docx]

**S1 Appendix**

**PRODOSE trial guidance for operating theatre and ICU management of bleeding and coagulopathy**

*Fig 1* Guidance for the intra- and post-operative management of coagulopathy and bleeding in patients enrolled in the PRODOSE trial. Assumes that clinically significant bleeding as judged by the cardiac anaesthesist, cardiac surgeon or intensivist is present. Use of the algorithm was encouraged until 24 hours postoperatively. ACT, activated clotting time; TEG, thromboelastography.

| **Blood product** | **Parameter for administration** |
| --- | --- |
| Packed red blood cells | Hb < 80 g/L |
| Fresh frozen plasma or prothrombin complex concentrate | r-time > 8 minutes, aPTT > 40 seconds, INR > 1.3 |
| Platelets | Platelet count < 100 × 10^9^/L, MA < 50 mm if functional fibrinogen assay > 20 mm |
| Cryoprecipitate or fibrinogen concentrate | Fibrinogen < 1.2 g/L, functional fibrinogen assay < 20mm if MA < 50 mm |

*Table 1* Guidelines for the administration of blood and blood products for patients enrolled in the PRODOSE trial based on point-of-care thromboelastography and laboratory studies. Assumes that clinically significant bleeding as judged by the cardiac anaesthetist, cardiac surgeon or intensivist is present. Use of the guidelines is required until 24 hours postoperatively. MA, maximum amplitude (TEG).
