# Supplementary material for: Optimal protamine dosing after cardiopulmonary bypass: The PRODOSE adaptive randomised controlled trial
Source: PLoS Med. 2021 Jun 7;18(6):e1003658. doi: 10.1371/journal.pmed.1003658 (PMC8216535; doi:10.1371/journal.pmed.1003658)
Supplement: S2 Appendix — (DOCX) [file pmed.1003658.s002.docx]

**S2 Appendix**

**PRODOSE trial supplemental methods**

**Response adaptive randomisation ratio**

Randomisation ratio was adapted at the interim analysis according to the following principles:

$$R^{*} =\left\{ \begin{matrix} \frac{s_{C}\sqrt{\bar{X}_{E}}}{s_{C}\sqrt{\bar{X}_{E}}+s_{E}\sqrt{\bar{X}_{C}}} & (\bar{X}_{C}<\bar{X}_{E}\text{ and }R^{+}>1\text{ or }\bar{X}_{E}<\bar{X}_{C}\text{ and }R^{+}<1) \\ \frac{1}{2} & \text{otherwise} \end{matrix} \right.$$

Where:

$$R^{+}=\frac{S_{C}\sqrt{\bar{X}_{E}}}{S_{E}\sqrt{\bar{X}_{C}}}$$

The ratio (R*) proposed by Zhang and Rosenbeger is only valid under strict assumptions of asymptotic normality in the primary endpoint ([1]), in which case it is expected to maximise allocations to a superior arm while preserving power. In simulations at the design stage, it was noted that in case of deviations from normality the randomisation change was not triggered even if the presence of an observed superiority of the treatment arm. To better include the case of deviations from normality in the design allowed the ratio to be adapted if the observed treatment difference at the interim was half the predicted difference (7.5%). The R* would then be set to 1:1.33 in favour of the beneficial treatment arm. Simulation studies showed that this value was in the 85% percentile of the randomisation ratios triggered with the original adaptive design and under the alternative hypothesis of a treatment effect. This threshold was chosen because the resulting power of the adaptive design under assumptions of exponential distributions resulted in 90% and the resulting allocations were similar in both cases.  Otherwise, the ratio would remain fixed and equal and the trial will not adapt after the interim. The above modification to the original design by Zhang and Rosenberger has been agreed as reflecting the maximum deviation for ethical reasons that were acceptable and appealing to clinicians and statisticians of this trial.

**Efficacy and futility boundaries**

The pre-defined stopping boundaries for futility at the interim and efficacy at study completion were *u*_1_ = 1.8 and *e*_2_ = -1.3 respectively. These test statistics were computed as follows:

$\text{t}_{\text{i}}= \frac{\bar{y}_{Ei}-\bar{y}_{Ci}}{{2S}_{i}\sqrt{{n_{i}}^{-1}}},\text{ }\text{i}=1,2$ S_i_ and n_i_ are the sample standard deviation and sample size at the i^th^ analysis. These stopping rules meant that if the treatment group has a mean r-time 20% higher than the control, the trial stops in its first interim with a 90% probability (that is out of 10000 simulated trials, 9000 stopped at 1st stage). If the treatment group has a mean r-time 14% higher than the control, the trial stops in its first interim with a 64% probability (again out of 10000 simulated trials). Under the null, the trial stops for futility in 3.4% of simulated trials and under the alternative this happens with 0 probability. If the primary outcome followed a non-normal distribution the efficacy analysis was be carried out using a resampling method.

**Resampling analysis**

Due to the non-normal nature of the data the primary efficacy analysis was carried out in resampling-based framework instead of the efficacy boundary. A randomisation-based approach ensured type I error is preserved despite any deviations in assumptions and taking the adaptive design into account ([2]). Patients were ordered by recruitment date and then their group assignment were resampled at random according to the original design. For the first 114 cases this was at a 1:1 ratio representing the first stage of the trial. At this point the effect size was assessed having noted a greater than 7.5% difference in treatment arms, the second 114 group assignments was sampled at a ratio of 1:1.33. If a difference of this magnitude had not been noted, the ratio would have remained at 1:1. This procedure was repeated 100,000 times and the t statistic was calculated for each iteration. Finally, we calculated the true p-value of the observed data as the proportion of times that there was a more extreme test statistic generated by chance.

**References**

1. Zhang L, Rosenberger WF. Response-adaptive randomization for clinical trials with continuous outcomes. Biometrics. 2006;62: 562–569. doi:10.1111/j.1541-0420.2005.00496.x

2. Simon R, Simon NR. Using randomization tests to preserve type I error with response adaptive and covariate adaptive randomization. Stat Probab Lett. 2011;81: 767–772. doi:10.1016/j.spl.2010.12.018
