# Supplementary material for: Optimal protamine dosing after cardiopulmonary bypass: The PRODOSE adaptive randomised controlled trial
Source: PLoS Med. 2021 Jun 7;18(6):e1003658. doi: 10.1371/journal.pmed.1003658 (PMC8216535; doi:10.1371/journal.pmed.1003658)
Supplement: S1 Protocol — (PDF) [file pmed.1003658.s003.pdf]

Papworth Hospital
NHS Foundation Trust

Title: PRODOSE TRIAL

**Study Title**	Algorithmic Protamine Dosing for Reversal of Heparin after Cardiopulmonary Bypass (PRODOSE)	
**Chief investigator**	Dr Florian Falter<br>Department of Anaesthesia and Intensive Care<br>Papworth Hospital<br>Cambridge CB23 3RE<br>UK	
**Trial Sponsor**	**Papworth Hospital NHS Foundation Trust**<br>Research & Development Unit, Papworth Hospital, Cambridge CB23 3RE<br>Tel: 01480 364143 Fax: 01480 364550	
**Trial Funder**		
**Co-investigators**	Name	Site
	Dr Lachlan Miles	Department of Anaesthesia<br>Austin Health<br>145 Studley Road<br>Heidelberg, VIC 3084<br>Australia
	Dr Christiana Burt	Department of Anaesthesia and Intensive Care<br>Papworth Hospital<br>Cambridge CB23 3RE<br>UK
	Dr Joe Arrowsmith	Department of Anaesthesia and Intensive Care<br>Papworth Hospital<br>Cambridge CB23 3RE<br>UK
	Dr Martin Besser	Pathology Department<br>Blood Sciences<br>Papworth Hospital<br>Cambridge CB23 3RE<br>UK
	Mr Ravi de Silva	Department of Surgery<br>Papworth Hospital<br>Cambridge CB23 3RE<br>UK
	Dr Sofia Villar	MRC Biostatistics Unit<br>Cambridge, CB2 0SR<br>UK
	Ms Carol Freeman	Papworth Trials Unit Collaboration<br>Papworth Hospital<br>Cambridge CB23 3RE<br>UK
**Study location**	Multicentre	Papworth Hospital NHSFT<br>Austin Health, Australia

Papworth Hospital
NHS Foundation Trust

Lay Summary

Open-heart surgery is routinely conducted using a heart-lung machine. In order to conduct operations involving heart-lung machines a patient's coagulation system needs to be reliably supressed to avoid clot formation. Clot in the extracorporeal circuit generally has fatal consequences.

In the vast majority of cases (>99%) the desired suppression of the blood clotting system is achieved by administering heparin. Although relatively short acting, with a half-life of about 150min for a full adult dose, heparin needs to be reversed after weaning from the heart-lung machine in order to avoid catastrophic bleeding post-operatively.

Heparin reversal is achieved by using protamine. This drug is derived from salmon sperm and is generally safe to use. However, in a reasonable number of cases it can have severe side effects, ranging from dangerous hypotension to high blood pressure in the lung circulation with adequately oxygenate the patient. Severe anaphylactic reactions have also been described. There is also increasing evidence that inadequately high doses of protamine may lead to an increased bleeding tendency.

There is controversy about the right dosing of protamine. Traditionally a pragmatic and empirical '1:1' formula is used reversing 100U of heparin with 1mg of protamine. This dosing regime does not take the decay of heparin during the time spent on the heart-lung machine into account and potentially exposes patients to unnecessarily high doses of protamine.

Our group was previously able to demonstrate in a pilot project that using a pharmacokinetic algorithm, which takes heparin decay into account, is able to reduce the protamine dose given to patients without increased bleeding or transfusion requirements.

We have continued to develop this algorithm into a 2 compartmental model and are seeking to test the hypothesis that using our formula can reduce patients' risk of the unwanted side-effects of protamine by reducing its dose.

1 STUDY SYNOPSIS

**Title**	Algorithmic Protamine Dosing for Reversal of Heparin after Cardiopulmonary Bypass (PRODOSE)
**Sponsor**	Papworth Hospital NHS Foundation Trust
**Medical condition**	Cardiac disease requiring cardiac surgery on cardiopulmonary bypass
**Purpose**	If the hypothesis of this study is proven, it will identify a new method for reducing blood loss and transfusion requirement after cardiac surgery, and provide a low cost alternative to existing heparin concentration devices.
**Primary objective**	Return to normal coagulation after cessation of cardiopulmonary bypass and reversal of heparin
**Secondary objectives**	Intercostal drain output 4 hours post-surgery<br>Use of blood products 24 hours post-surgery
**Trial design**	A prospective, two-centre, double-blinded, two-stage, randomised controlled trial.
**Study Endpoints**	**Primary**<br>Return to normal thrombelastography profile<br>**Secondary**<br>Blood loss and blood product usage post-surgery
**Sample size**	200
**Eligibility criteria**	***Inclusion Criteria:***<br>Patients scheduled to undergo elective cardiac surgery<br><br>***Exclusion Criteria:***<br>Emergency surgery<br>Age < 18 years<br>Known or suspected coagulopathy or platelet dysfunction<br>ADP-receptor antagonists within 7 days of surgery (clopidogrel, ticlodipine, pasugrel)<br>Total body weight > 130kg<br>End stage renal failure requiring dialysis<br>Plan for severe hypothermia (< 28°C) or deep hypothermic circulatory arrest<br>Complex cardiac surgery (redo sternotomy, surgery on the thoracic aorta [excluding root])<br>Transplantation

**Screening and Enrolment**	Patients will be identified on the day before surgery is scheduled, once the operating list has been published. If required by the REC patients will be consented on the evening before, or day of surgery for same day admissions.
**Baseline & Randomisation**	Sealed Envelope (an internet based company specialised in providing randomisation services to trials) will produce the group allocation under the instructions of PTUC and Dr Villar. Randomisation ratio will be updated after the interim analysis to favour a superior arm whilst preserving the power levels.
**Interventions**	No additional interventions are required to standard practice apart from adjusting protamine dose in the treatment group
**Follow up**	No long term follow-up is planned.
**End of Study**	Once number of patients estimated by power calculation has been included. It is expected that patient recruitment is completed within 9 – 12 months
**Procedures for safe monitoring**	A futility stopping rule is included to stop the trial – if necessary - after 50% recruitment and treatment.
**Criteria for modifying or discontinuing allocated intervention**	No modifications are planned; however the study will be abandoned if a significant increase in blood product usage is demonstrated in the algorithm group.

Papworth Hospital
NHS Foundation Trust

TABLE OF CONTENTS

1 Study Synopsis ... 3
2 Introduction ... 7
2.1 Background ... 7
2.2 Rationale ... 7
2.3 Expected Output of Research/Impact ... 9
3 Trial Objectives ... 10
3.1 Primary Objective ... 10
3.2 Secondary Objectives ... 10
3.3 Study End Points ... 10
3.3.1 Primary Endpoint ... 10
3.3.2 Secondary Endpoints ... 10
4 Trial Design ... 11
4.1 Statement of design ... 11
4.2 Study Setting ... 11
4.3 Study protocol ... 12
4.4 Sample Size ... 14
5 Participant Recruitment, Randomisation and Follow up ... 16
5.1 Study Population and eligibility ... 16
5.2 Participant identification and informed consent procedure ... 16
5.3 Randomisation ... 17
5.4 interventions ... 17
5.5 Participant follow up ... 17
6 Data Handling and Record keeping ... 19
6.1 Screening and recruitment ... 19
6.2 Baseline and clinical follow up data ... 19
6.2.1 Primary Endpoint ... 20
6.2.2 Secondary Endpoints ... 20
6.3 Recording and management of Adverse Events ... 20
7 Statistics ... 20
8 Project Management ... 20
8.1 Research Management and governance ... 20
8.2 Study Registration ... 21

8.3 Trial Steering Committee (TSC) ... 21
8.4 Data Monitoring Committee (DMC) ... 21
8.5 CRN Eastern ... 21
9 Ethical & Research Governance approvals UK sites ... 21
9.1 Initial REC and HRA Approval ... 21
9.2 Site Capability and Capacity ... 21
9.3 Protocol amendments ... 22
10 Insurance ... 22
11 Publication Policy ... 22
12 References ... 22
13 Appendix 1: Definitions of Adverse Events ... 25
13.1 Adverse Event (AE) ... 25
13.2 Serious Adverse Event ... 25
13.3 Expected morbidity ... 25
13.3.1 Expected morbidity following PRODOSE intervention: ... 25

2 INTRODUCTION

2.1 BACKGROUND

Systemic, high-dose anticoagulation (prevention of blood clotting) is fundamental to the safe conduct of cardiopulmonary bypass (CPB). Globally, unfractionated heparin remains the most widely used agent for this purpose. It is found on the WHO Model List of Essential medications, reflecting affordability, predictable behaviour when being metabolised, and the availability of an effective reversal agent: protamine. However, where heparin is lauded for its ease of use, the administration of protamine is undertaken cautiously, due to its well-known side-effect profile. Where much of the early focus in the literature was on the negative cardiovascular effects of the drug, attention has recently turned to its anticoagulation properties, particularly in excessive doses, and with special implications for preventing bleeding after CPB.

The continued use of relatively high, fixed protamine dosing for the reversal of heparin has been increasingly questioned in the literature[14-17] due to increasing recognition of substantial variability between individuals. This has led to the development of heparin concentration devices designed to better predict protamine requirements during cardiac surgery. However, such devices are often expensive, and the benefits of using them have been questioned. The last few years have bought the development of statistical and pharmacokinetic models for protamine administration as an alternative[20-23].

Our group has developed a dual compartment pharmacokinetic algorithm that can be tailored to the individual patient using ideal body weight, and the doses and time intervals of administered heparin before and during CPB. This algorithm is already in use locally, nationally and internationally following the publication of promising pilot data[28] and similar data from a Dutch group[21].

2.2 RATIONALE

1. Transfusion after cardiac surgery

It has been recognised that the transfusion of allogeneic blood, despite the improvements in oxygen delivery that it imparts, has multiple negative physiological consequences. Transfusion has been shown to be associated with increased morbidity related to ischaemia,[1] infection,[2–5] renal impairment,[6] post-CABG graft occlusion[7] and acute lung injury.[8] With respect to longer term outcomes, Engoren and colleagues found that blood transfusion during cardiac surgery was associated with a doubling of the risk of death at 5 years.[9] Whilst the aetiology of these reactions is not yet fully defined, it almost certainly relates to the presence of allogeneic leukocyte material, despite the universal leukoreduction of packed red blood cells, and associated transfusion related immunomodulation. This phenomenon results in a down regulation of host immune response, and consequently, allogeneic red cell transfusion has been shown to be a strong predictor of all-cause mortality after cardiac surgery.

Yet despite the host of negative consequences associated with transfusion following cardiac surgery, recent evidence has suggested that allowing severe anaemia to develop before transfusion is not a better alternative. In a recent large, randomised controlled trial,[10] a restrictive transfusion threshold of < 7.5g.dl^{-1} was compared with a more liberal threshold of < 9 g.dl^{-1}. Surprisingly (given what was thought to be known about allogeneic transfusion in this population), there were more deaths in the restrictive-threshold group than in the liberal-threshold group (4.2% vs. 2.6%; hazard ratio, 1.64; 95% CI, 1.00 to 2.67; P = 0.045). This study has substantially influenced the body of literature on transfusion, with recent meta-analyses suggesting that the overall preponderance of evidence is turning in favour of transfusion.[11]

This finding creates more difficulties. Transfusing more patients would incur a substantial cost on the health system, both with respect to the costs of procuring and processing blood products, and the implications of a further strain on an already scarce and expensive resource. Attention must turn, therefore, to strategies to prevent any transfusion threshold, liberal or restrictive, from being reached in the first place. Minimising or preventing intra-operative and post-operative blood loss most effectively achieves this.

2. Excessive protamine administration and haemostatic effects

Systemic, high-dose anticoagulation is fundamental to the safe conduct of cardiopulmonary bypass (CPB). Globally, unfractionated heparin remains the most widely used agent for this purpose[12,13]. It is found on the WHO Model List of Essential medications, reflecting affordability, predictable pharmacokinetics, and the availability of an effective reversal agent: protamine. However, where heparin is lauded for its ease of use, the administration of protamine is undertaken cautiously, due to its well-known side-effect profile. Where much of the early focus in the literature was on the negative cardiovascular effects of the drug[14], attention has recently turned to its anticoagulation properties[15–17], particularly in excessive doses, and with special implications for haemostasis after CPB.

At present, protamine is normally dosed at a fixed ratio, based on the dose of heparin required to establish therapeutic anticoagulation prior to bypass. The most frequently used dose is referred to as 1:1, or 1mg of protamine for every 100 IU of heparin that was required to establish anticoagulation.

It has long been recognised that protamine is an anticoagulant in its own right. Excessive dosing downregulates thrombin generation through inhibition of factor V[17] and platelet aggregation.[16] Clinical evidence now exists that excessive protamine dosage increases the risk of post-operative bleeding, whereby a high fixed dose ratio of protamine (1.3:1) compared to a lower fixed dose ratio (0.8:1) led to higher intrinsic clotting times, lower thrombin generation and higher post-operative blood loss.[18]

In preparation of this proposal a retrospective analysis of a prospectively collected database was undertaken at Austin Health, reviewing all cases of on-pump coronary artery bypass grafts and single valve repair/replacements from 01/01/2011 to 31/12/2015. Using a moderate protamine:heparin (0.6 – 1:1) dosage group as a reference range, the low dose protamine:heparin (< 0.6:1) group was 56.46% less likely to receive a PRBC transfusion (OR 0.435; 95% CI 0.270 – 0.703 p = 0.001) while the high dose group (> 1:1) carried a 241% increased risk of transfusion (OR 3.412; 95% CI 2.399 – 4.853 p < 0.001).

3. The PRODOSE algorithm

Whilst it has been established that excessive protamine dosage increases the risk of post-operative bleeding and transfusion, it is also true that inadequate reversal of heparin also increases post-operative bleeding through the perpetuation of anticoagulant effects. This begs the question whether or not fixed dose ratios (even in the setting of lower doses of protamine) are the most effective strategies in reversing heparin. This is perhaps best highlighted by the considerable interindividual variability in heparin requirements that can be demonstrated during CPB.[19]

In light of this, further researches are beginning to appear in the literature advocating the use of statistical or pharmacokinetic models for the dosing of protamine in cardiac surgery[20–23] In a collaboration with Austin Health and the University of Melbourne in Australia, and Papworth Hospital NHS Foundation Trust in the UK, we have developed a dual compartment model that predicts heparin concentration over the course of cardiopulmonary bypass and permits precise dosing of protamine when reversal is required.

In a small pilot study, 60 patients were allocated to receive either protamine dosed according to an earlier, single compartment model of the aforementioned algorithm ($n = 30$), or using conventional treatment with a fixed dose ratio of 1:1 ($n = 30$). Patients who had protamine dosing according to the algorithm demonstrated a lower protamine requirement post-bypass relative to empirical management as measured by absolute dose (243 ± 49mg vs. 305 ± 34.7mg; $p < 0.001$) and heparin to protamine ratio (0.79 ± 0.12 vs. 1.1 ± 0.15; $p < 0.001$). There was no difference in the pre- to post-bypass ACT ratio (1.05 ± 0.12 vs. 1.02 ± 0.15; $p = 0.9$). Whilst lower ICC outputs and allogeneic transfusion rates were seen in the algorithm group, these differences were not statistically significant[28].

2.3 Expected Output of Research/Impact

This study will aim, with appropriate power, to prospectively determine if the use of a bespoke pharmacokinetic algorithm is superior in returning coagulation parameters to normal, pre-heparin values, thereby preventing bleeding and subsequent requirement for blood transfusion post-operatively when compared with standard practice – the use of a fixed dose of protamine. If the hypothesis is shown to be right this is likely to impact internationally on the practice of reversing full heparinisation after cessation of cardiopulmonary bypass.

3 Trial Objectives

3.1 Primary Objective

We aim to demonstrate that the use of a bespoke pharmacokinetic algorithm can more reliably return coagulation parameters to pre-heparin levels, decrease the risk of post-operative bleeding and transfusion through superior titration of protamine after cardiopulmonary bypass

3.2 Secondary Objectives

As previously noted, the recognition of the negative coagulation effects of protamine and inter-individual variability is not adequately accounted for by fixed dose ratios, while alternatives such as heparin concentration devices are relatively expensive.

Recently attention has turned to pharmacokinetic algorithms to bridge the gap in practice. Up until now, any field-testing of such algorithms has been done in an in-vitro, retrospective or non-randomised fashion. The PRODOSE study will represent the first RCT in this evolving area, and has the potential to substantially influence practice and policy, specifically, that where a heparin concentration device is not available, consideration should be given to using a pharmacokinetic algorithm rather than fixed dosing ratios.

3.3 Study End Points

3.3.1 Primary Endpoint

- Kaolin TEG r-time at 3 minutes post-protamine administration after cessation of cardiopulmonary bypass.

3.3.2 Secondary Endpoints

- Kaolin ACT: Heparinase ACT ratio at 3 minutes after protamine administration;
- Kaolin ACT pre-bypass : Kaolin ACT post-bypass ratio at 3 minutes after protamine administration;
- Kaolin TEG r-time pre-bypass: Kaolin TEG r-time post-bypass ratio;
- Intercostal catheter drainage at 4 hours post-operatively;
- Allogeneic blood transfusion 24 hours post-operatively.

4 TRIAL DESIGN

4.1 STATEMENT OF DESIGN

A prospective, two-centre, double-blinded, two-stage, randomised controlled trial.

4.2 STUDY SETTING

The PRODOSE algorithm

Initially our group our group was mainly concerned with the derivation of an exponential decay curve for the central pharmacokinetic department. This was accomplished by deriving a time constant (*k*) based on ideal body weight from publicly available pharmacokinetic data[27].

Residual heparin (and thus the protamine dose) is calculated using the dose of heparin administered to achieve anti-coagulation sufficient for the safe conduct of CPB, and the ideal body weight of the patient. This information is then used to derive an exponential decay curve.

An exponential decay curve is based on the equation:

$$H = Ae^{-kt}$$

Where *H* represents the amount of heparin in the system at point t, *A* represents the amount of heparin in the system at time zero, *k* represents the reaction time constant and *t* represents the time elapsed since the dose of heparin was administered.

To determine the individual heparin metabolic pattern of the patient, *k* must be derived. To do so, we must determine *H* at a specific point in time (*t*).

Heparin half-life ($t^{\frac{1}{2}}$) is derived by:

$$t^{\frac{1}{2}} = 26 + 0.323\left(\frac{Heparin\ in\ system}{Ideal\ Body\ Weight}\right)$$

Assuming an initial heparin amount of 1 (100%), the amount remaining once one half-life has elapsed is 0.5 (or 50% the original amount of heparin).

$$\therefore 0.5 = e^{-kt}$$

$$\therefore 0.5 = e^{-kt^{\frac{1}{2}}}$$

$$-k = \frac{log_e 0.5}{t^{\frac{1}{2}}}$$

$$\therefore k = -\frac{log_e 0.5}{t^{\frac{1}{2}}}$$

Once the exponential decay curve has been derived, the amount of heparin within the system at any point in time can be calculated, based on the original loading dose. Subsequent doses of heparin (administered if the ACT falls below the safe range) are added to the total, with the time elapsed since the previous dose allowing the residual dose of heparin remaining to be derived.

At the conclusion of CPB, the final amount of heparin within the system is calculated, and a protamine dose sufficient to reverse this amount is administered at a ratio of 1:1.

Subsequent to these results, we have collaborated with statisticians at the University of Melbourne to devise a second compartment for the algorithm, using data from another trial in this area[21]. Rather than endeavouring to correct for patient body habitus, Meesters et al elected to use a common approach between all patients as per the standard equation for first order pharmacokinetics, whereby:

$$C_t = (C_0 \times A \times e^{-\alpha t}) + (C_0 \times B \times e^{-\beta t})$$

Whereby A = 0.1, α = 10 (or K_{12}), B = 0.9, β = 250 and C_0 reflects the initial dose of heparin. By combining the two equations (the central compartment of our formula with the peripheral compartment of the Meesters formula), we yield the following algorithm:

$$C_t = (C_0 \times A \times e^{-\alpha t}) + \left(C_0 \times B \times e^{-\left[\frac{\ln 0.5}{26+0.323\left(\frac{Heparin\ in\ system}{Ideal\ Body\ Weight}\right)}\right]}\right)$$

4.3 Study Protocol

The majority of patients will be identified on the day before surgery.

Patients will be initially randomised with an equal probability using a block-randomisation procedure to receive either a fixed dose ratio of protamine or dosing according to the aforementioned algorithm. After the interim analysis, if the trial is not stopped for futility[30], the randomisation ratio will be updated in order to optimise response without compromising the power of the study[31].

As is routine,

- a baseline heparinase and kaolin ACT and TEG will be taken;
- standard practice for the initiation and management of anticoagulation during cardiopulmonary bypass will be followed;
- heparin will be administered at surgical request at a dose of 300 $IU.kg^{-1}$, and cardiopulmonary bypass initiated once ACT reaches a predefined safe level (400 – 450 seconds);
- an additional 5,000 IU of heparin will be included in the bypass circuit prime;
- repeat ACT measurements will be taken every 30 minutes during bypass and further heparin administered at the discretion of the case anaesthetist and the clinical perfusionist to maintain an ACT > 400 seconds.

As per usual routine, at the conclusion of cardiopulmonary bypass protamine will be given at surgical request. For patients in the control group, protamine will be dosed on a 1:1 ratio

according to the total dose of heparin initially required to establish a therapeutic ACT (i.e. if 30,000 IU were required prior to initiating cardiopulmonary bypass, then the protamine dose will be 300mg). For patients in the intervention group, protamine will be administered according to the PRODOSE algorithm, which has been incorporated into an Excel spreadsheet for ease of use (Microsoft Corporation, Redmond, WA). The protamine dose will be administered at the anaesthetists' discretion.

Three minutes after protamine administration, blood samples will be taken for ACT and TEG, FBE and coagulation profile. If kaolin ACT has returned to within 10% of baseline, no further protamine will be administered. If the kaolin ACT is > 10% above baseline, or the surgeon complains of excessive microvascular bleeding, then the algorithm in Figure 1 will be used to facilitate correction of this.

In the event that residual pump blood is administered to the patient after the conclusion of bypass a dose of protamine equivalent to 10% of the total initial dose for every 500mL will be administered (i.e. if initial dose of protamine is 200mg, and 1000mL of residual blood is infused then the dose administered with the pump blood will be 40mg).

Further blood products will be administered (both in theatre and in ICU) in accordance with the Papworth protocol for usage of blood products if the following limits are reached and bleeding is judged to be excessive by attending medical staff:

Product	*Parameter for administration*
Packed red blood cells	Hb < $70 g.L^{-1}$
Fresh frozen plasma	r-time > 8 minutes, aPTT > 40 seconds, INR > 1.3
Platelets	Platelet count < $100 \times 10^{12}.L^{-1}$, MA < 50mm if functional fibrinogen assay > 20mm
Fibrinogen	Fibrinogen < $1.2 g.L^{-1}$, functional fibrinogen assay < 20mm if MA < 50mm

Further data will be collected regarding ICC drainage in ICU, and requirement for allogeneic transfusion. This information will be prospectively collected from the medical record.

4.4 SAMPLE SIZE

Study size has been determined from the 1:1 population reported in Meesters et al[21] using the data recorded for the relevant coagulation parameters (r-time) after protamine administration.

**Measure**	**Value**
Mean r-time (control)	250 seconds
Mean r-time (intervention)	214.5 seconds *(15% decrease)*
Standard deviation	76 seconds
α	0.05
β	0.90
Sample size	88

Taking into account loss of data (due to failure to follow up, equipment failure etc.), we aim to recruit 100 patients into each of the two study groups for a total of 200 patients. The type I error rate and the power of the study for the sample size were calculated through simulations (although a close form approximation would also be available).

Figure 1: Protocol for post-bypass coagulopathy

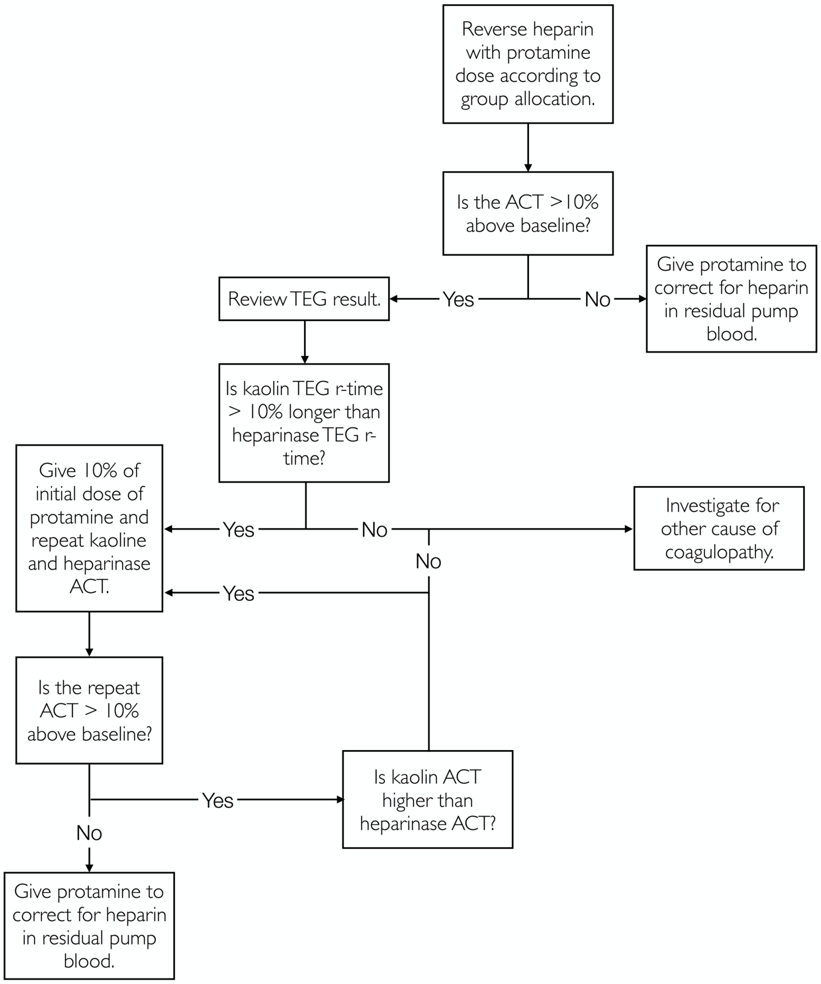

5 Participant Recruitment, Randomisation and Follow up

5.1 Study Population and eligibility

Inclusion Criteria:
Patients scheduled to undergo elective cardiac surgery on cardiopulmonary bypass
Exclusion Criteria:
Emergency surgery
Age < 18 years
Known or suspected coagulopathy or platelet dysfunction
ADP-receptor antagonists within 7 days of surgery (clopidogrel, ticlodipine, pasugrel)
Total body weight > 120kg
End stage renal failure requiring dialysis
Plan for severe hypothermia (< 28°C) or deep hypothermic circulatory arrest
Complex cardiac surgery (redo sternotomy, surgery on the thoracic aorta [excluding root])
Solid Organ Transplantation

5.2 Participant identification and informed consent procedure

Screening for eligibility criteria and consenting to the study will be done by members of the care team. A member of the direct care team will take consent as part of obtaining consent for surgery and anaesthesia.

Patients will be identified from the operating list when published on the day before planned surgery. This will allow us to exclude the following criteria:

- Emergency surgery
- Age < 18 years
- End stage renal failure requiring dialysis
- Plan for severe hypothermia (< 28°C) or deep hypothermic circulatory arrest
- Complex cardiac surgery (redo sternotomy, surgery on the thoracic aorta [excluding root])
- Solid Organ Transplantation

Reviewing notes will inform on the remaining exclusion criteria:
- Known or suspected coagulopathy or platelet dysfunction
- ADP-receptor antagonists within 7 days of surgery (clopidogrel, ticlodipine, pasugrel)
- Total body weight > 120kg

Eligible patients will be given a written information sheet and will be talked through the project. They will be given ample opportunity to ask questions and voice their concerns before giving / refusing informed consent.

Patients will be consented on the afternoon on the day before surgery. They will again be asked about participation during the check-in process into theatre and can withdraw their consent up until induction of anaesthesia, which will happen on average between 16 - 24 hours after consenting.

5.3 Randomisation

Randomisation process
Randomisation will be done either web or telephone based in accordance with Papworth R&D procedures taking the two-site, two continent nature of the trial into consideration. Sealed Envelope (an internet based company specialised in providing randomisation services to trials) will produce the group allocation under the instructions of PTUC and Dr Villar.

Randomisation issues
In recent months both Papworth Hospital NHS Foundation Trust and Austin Health have seen an increase in delayed or cancelled operations due to pressures on the service. To avoid patients being randomised and subsequently not being included because their operation is cancelled it is prudent to only randomise once a patient is in the anaesthetic room, about to be anesthetised.

5.4 Interventions

At the conclusion of cardiopulmonary bypass, protamine administration will be undertaken at surgical request. For patients in the control group, protamine will be dosed on a 1:1 ratio according to the total dose of heparin initially required to establish a therapeutic ACT (i.e. if 30,000 IU were required prior to initiating cardiopulmonary bypass, then the protamine dose will be 300mg). For patients in the intervention group, protamine will be administered according to the PRODOSE algorithm, which has been incorporated into an Excel spread sheet for ease of use (Microsoft Corporation, Redmond, WA).

Criteria for modifying or discontinuing allocated intervention

After the first 50% of patients have been recruited and treated an interim analysis will take place. A formal futility stopping rule will be binding and will ensure that the operating characteristics of the trial (type I error and power) are preserved. If the trial progresses into the second phase the allocation ratio will be updated in order to optimise response without compromising the power of the study[31]

5.5 Participant follow up

There is no follow up planned beyond 24 hours post-operatively

Table 1: Schedule of Events

**Specific Activity**	**Undertaken by**	**Screening**	**Baseline Rando-misation**	**Intra-OP**	**ICD output 4h post-OP**	**Blood product usage 24h post-OP**
Identify potential participant	Local MDT	x				
Eligibility check (exclusions)	Local MDT	x				
Approach potential participant to discuss study	Local PI	x				
Take informed consent	PI or Co-investigator		x			
Baseline clinical data collection, including inclusion and exclusion criteria	Local Research Nurse		x			
Randomisation (web or telephone)	Local Research Nurse		x			
Operation Included patients	PI or Co-investigator			x		x
Clinical Follow up data	Local Research Nurse			x	x	x
Health Service and Resource use data	MDT			x	x	x

6 DATA HANDLING AND RECORD KEEPING

The trial will be conducted according to the Good Clinical Practice and Standard Operating Procedures of Papworth Trials Unit Collaborative (PTUC) to ensure the monitoring and safety of trial participants and data validity.

6.1 SCREENING AND RECRUITMENT

Patients will be identified and recruited by the clinical care team, with support from a Research Nurse or Clinical Trial Coordinator.

6.2 BASELINE AND CLINICAL FOLLOW UP DATA

Baseline data will be include:

- Patient demographics
- Type of surgery
- EuroSCORE
- Pre-OP medication, particularly anticoagulants
- Pre-OP routine lab (Full Blood Count, coagulation profile)

Intraoperative data will include:

- Routine intra-OP lab at standard time points (arterial blood gasses, ACT)
- Heparin dose
- Protamine dose
- Cardiopulmonary bypass time
- Aortic cross-clamp time
- Lowest temperature
- Thrombelastography result

Follow up Data will be include:

- Post-operative routine lab (FBE, coagulation profile)
- Post-operative intercostal chest tube drainage
- Post-operative allogeneic transfusion (Red Blood Cells, Fresh Frozen Plasma, Platelets, Factor concentrates)
- Post-operative additional protamine doses

6.2.1 Primary Endpoint

- Kaolin TEG r-time at 3 minutes post-protamine administration after cessation of cardiopulmonary bypass.

6.2.2 Secondary Endpoints

- Kaolin ACT: Heparinase ACT ratio at 3 minutes after protamine administration;
- Kaolin ACT pre-bypass : Kaolin ACT post-bypass ratio at 3 minutes after protamine administration;
- Kaolin TEG r-time pre-bypass: Kaolin TEG r-time post-bypass ratio;
- Intercostal catheter drainage at 4 hours post-operatively;
- Allogeneic blood transfusion 24 hours post-operatively.

6.3 Recording and management of Adverse Events

No Serious Adverse Events (SAE) are expected. A previous pilot investigation has demonstrated that the trial design in both feasible and safe. There is no deviation from standard clinical practice in order to facilitate the trial.

All SAE occurring between randomisation and the end of follow-up will be recorded in the patient's hospital notes and submitted, within 24 hours of the site becoming aware, to Papworth Trials Unit Collaboration using an SAE form.

All recorded SAEs will be reported to the Sponsor and the Data Monitoring Committee (DMC). If an SAE occurs that is considered to be both unexpected and related to the study protocol (SUSAR), it will be reported within 24 hours of recognition.

Non-serious Adverse Events will be not be recorded or reported for the PRODOSE trial, unless they form part of the clinical event dataset.

The Sponsor will report any SUSARs to the Research Ethics Committee within 15 days of their knowledge of the event and local PIs will be notified.

Details of Expected Adverse Events are listed in Appendix 1.

7 Statistics

Analysis of the primary outcome will include all patients. Numerical data will be analysed using an appropriate test based on the asymptotic distribution of the test statistic computed for the two-stage design. The chi-squared test or Fisher's exact test will be used for categorical data. Data will be reported using confidence intervals and p-values where appropriate.

8 Project Management

8.1 Research Management and governance

The Senior R&D Manager based at PTUC will oversee the study.

The Trial Manager(s) will co-ordinate all trial-related activities across the participating sites, monitor progress against the project milestones and manage the finances.

Data management activities will be carried out by the investigators, trial statistician and the Research Nurse/Clinical Trial Coordinator in collaboration with PTUC.

8.2 Study Registration

The study will be registered with an International Standard Randomised Controlled Trial Number (ISRCTN) and/or with ClinicalTrials.gov.

8.3 Trial Steering Committee (TSC)

A TSC responsible for day-to-day running of the study will meet at least every 3 months by teleconference to discuss recruitment, safety, data management and local site issues.

The TSC will comprise the Chief Investigator, co-applicants, the trial manager, statistician, data manager and representatives from each site.

8.4 Data Monitoring Committee (DMC)

As this is a relatively low risk study a DMC will not be convened unless requested by the funding committee.

8.5 CRN Eastern

Our primary linkage will be with Division 1 of the Eastern Clinical Research Network (CRN).

9 Ethical & Research Governance approvals UK sites

Similar approval will be sought for Austin Health before participation in keeping with Australian research legislation.

9.1 Initial REC and HRA Approval

The protocol and all patient-facing documentation will be submitted to a Research Ethics Committee (REC) and for Health Research Authority (HRA) approval prior to study commencement. HRA Approval is the process for the NHS in England that brings together the assessment of governance and legal compliance with the independent REC opinion provided through the UK research ethics service.

9.2 Site Capability and Capacity

HRA approval replaces the need for local checks of legal compliance and related matters by each participating organisation in England. This allows participating organisations to focus their resources on assessing, arranging and confirming their capacity and capability to deliver the

study. The Trial manager will work with the Sponsor to assist the local site with study set up in line with the HRA approval process.

9.3 Protocol amendments

Substantial amendments to the protocol and any patient-facing documentation will be submitted to a Research Ethics Committee (REC) and Health Research Authority for approval prior to implementation.

Amendments may only be implemented after a copy of the HRA approval letter has been obtained and local R&D departments have confirmed capacity to accommodate the amendment at that site.

Amendments intended to eliminate an immediate hazard to subjects may be implemented prior to receiving REC approval. However, in this case, approval must be obtained as soon as possible after implementation.

10 Insurance

UK Centres will be covered by NHS indemnity for negligent harm providing researchers hold a contract of employment with the NHS, including honorary contracts held by academic staff.

11 Publication Policy

The findings of this research will be disseminated by
- presentation at national and international cardiac anaesthesia conferences
- publication in a relevant journal such as Anesthesia & Analgesia or the Journal of Cardiothoracic and Vascular Anaesthesia

12 References

1. Murphy GJ, Reeves BC, Rogers CA, Rizvi SIA, Culliford L, Angelini GD. Increased mortality, postoperative morbidity, and cost after red blood cell transfusion in patients having cardiac surgery. *Circulation*. 2007;116(22):2544-2552. doi:10.1161/CIRCULATIONAHA.107.698977.
2. Horvath KA, Acker MA, Chang H, et al. Blood Transfusion and Infection After Cardiac Surgery. *Ann Thorac Surg*. 2013;95(6):2194-2201. doi:10.1016/j.athoracsur.2012.11.078.
3. Chelemer SB, Prato BS, Cox PM, O'Connor GT, Morton JR. Association of bacterial infection and red blood cell transfusion after coronary artery bypass surgery. *Ann Thorac Surg*. 2002;73(1):138-142.
4. Banbury MK, Brizzio ME, Rajeswaran J, Lytle BW, Blackstone EH. Transfusion increases the risk of postoperative infection after cardiovascular surgery. *J Am Coll Surg*. 2006;202(1):131-138. doi:10.1016/j.jamcollsurg.2005.08.028.
5. Murphy PJ, Connery C, Hicks GL, Blumberg N. Homologous blood transfusion as a risk factor for postoperative infection after coronary artery bypass graft operations. *J Thorac Cardiovasc Surg*. 1992;104(4):1092-1099.
6. Karkouti K. Transfusion and risk of acute kidney injury in cardiac surgery. *Br J Anaesth*.

2012;109(SUPPL1):29-38. doi:10.1093/bja/aes422.
7. Engoren M, Schwann TA, Jewell E, et al. Is transfusion associated with graft occlusion after cardiac operations? *Ann Thorac Surg*. 2015;99(2):502-508. doi:10.1016/j.athoracsur.2014.09.028.
8. Bux J. Transfusion-related acute lung injury (TRALI): a serious adverse event of blood transfusion. *Vox Sang*. 2005;89(1):1-10. doi:10.1111/j.1423-0410.2005.00648.x.
9. Engoren MC, Habib RH, Zacharias A, Schwann TA, Riordan CJ, Durham SJ. Effect of blood transfusion on long-term survival after cardiac operation. *Ann Thorac Surg*. 2002;74(4):1180-1186. http://www.ncbi.nlm.nih.gov/pubmed/12400765. Accessed August 4, 2016.
10. Murphy GJ, Pike K, Rogers CA, et al. Liberal or restrictive transfusion after cardiac surgery. *N Engl J Med*. 2015;372(11):997-1008. doi:10.1056/NEJMoa1403612.
11. Hovaguimian F, Myles PS. Liberal Transfusion Strategy in the Perioperative and Acute Care Settings. *Anesthesiology*. 2016;125(1):46-61.
12. Rosin MW, Holt D. Heparin Dose and Postoperative Bleeding in Patients Undergoing Cardiopulmonary Bypass. *J Extra Corpor Technol*. 2013;45(4):228.
13. Bouraghda A, Gillois P, Albaladejo P. Les différentes alternatives d'anticoagulation au couple héparine/protamine en chirurgie cardiaque sous circulation extra-corporelle. *Can J Anesth Can d'anesthésie*. 62(5):518-528. doi:10.1007/S12630-015-0339-6.
14. Holland L. Adverse Reactions to Protamine Sulfate Following Cardiac Surgery. 1984;162:157-162.
15. Khan NU, Wayne CK, Barker J, Strang T. The effects of protamine overdose on coagulation parameters as measured by the thrombelastograph. *Eur J Anaesthesiol*. 2010;27(7):624-627. doi:10.1097/EJA.0b013e32833731bd.
16. Mochizuki T, Olson PJ, Szlam F, Ramsay JG, Levy JH. Protamine reversal of heparin affects platelet aggregation and activated clotting time after cardiopulmonary bypass. *Anesth Analg*. 1998;87(4):781-785. doi:10.1213/00000539-199810000-00008.
17. Ni Ainle F, Preston RJS, Jenkins PV, et al. Protamine sulfate down-regulates thrombin generation by inhibiting factor V activation. *Blood*. 2009;114(8):1658-1665. doi:10.1182/blood-2009-05-222109.
18. Meesters MI, Veerhoek D, de Lange F, et al. Effect of high or low protamine dosing on postoperative bleeding following heparin anticoagulation in cardiac surgery. A randomised clinical trial. *Thromb Haemost*. 2016;116(2):251-261. doi:10.1160/TH16-02-0117.
19. Ranucci M, Isgrò G, Cazzaniga A, et al. Different patterns of heparin resistance: therapeutic implications. *Perfusion*. 2002;17(3):199-204. http://www.ncbi.nlm.nih.gov/pubmed/12017388. Accessed September 13, 2016.
20. Jia Z, Tian G, Ren Y, Sun Z, Lu W, Hou X. Pharmacokinetic model of unfractionated heparin during and after cardiopulmonary bypass in cardiac surgery. *J Transl Med*. 2015;13:45. doi:10.1186/s12967-015-0404-5.
21. Meesters MI, Veerhoek D, de Jong JR, Boer C. A Pharmacokinetic Model for Protamine Dosing After Cardiopulmonary Bypass. *J Cardiothorac Vasc Anesth*. 2016:1-6. doi:10.1053/j.jvca.2016.04.021.
22. Ödling Davidsson F, Johagen D, Appelblad M, Svenmarker S. Reversal of Heparin After Cardiac Surgery: Protamine Titration Using a Statistical Model. *J Cardiothorac Vasc Anesth*. 2015;29(3):710-714. doi:10.1053/j.jvca.2014.12.006.
23. Kjellberg G, Sartipy U, van der Linden J, Nissborg E, Lindvall G. An Adjusted Calculation Model Allows for Reduced Protamine Doses without Increasing Blood Loss in Cardiac Surgery. *Thorac Cardiovasc Surg*. 2016;64(6):487-493. doi:10.1055/s-0035-1558649.
24. Despotis GJ, Joist JH, Hogue CW, et al. The impact of heparin concentration and activated clotting time monitoring on blood conservation. A prospective, randomized evaluation in patients undergoing cardiac operation. *J Thorac Cardiovasc Surg*. 1995;110(1):46-54. doi:10.1016/S0022-5223(05)80008-X.
25. Despotis GJ, Gravlee G, Filos K, Levy J. Anticoagulation monitoring during cardiac surgery: a review of current and emerging techniques. *Anesthesiology*. 1999;91(4):1122-1151. doi:10.1097/00000542-199910000-00031.
26. Shore-Lesserson L, Reich DL, DePerio M. Heparin and protamine titration do not improve haemostasis in cardiac surgical patients. *Can J Anaesth = J Can d'anesthésie*. 1998;45(1):10-18.

doi:10.1007/BF03011985.

27. Brunton L, Chabner B, Knollman B, eds. Goodman & Gilman's The Pharmacological Basis of Therapeutics (12th Edition). In: *Goodman & Gilman's The Pharmacological Basis of Therapeutics (12th Edition)*. 12th ed. New York: McGraw Hill; 2011:1937.
28. Miles L, Machiori P, Falter F. Pilot validation of an individualised pharmacokinetic algorithm for protamine dosing after systemic heparinisation for cardiopulmonary bypass. epub ahead of print, DOI: 10.1177/0267659117697814 | First Published March 9, 2017
30. Jennison C, Turnbull B. Adaptive and nonadaptive group sequential tests. Biometrika. 2006; 93(1): 1-21.
31. Zhang L, Rosenberger W. Response-Adaptive Randomization for Clinical Trials with Continuous Outcomes. Biometrics. 2006; 62(2): 562-569.

13 Appendix 1: Definitions of Adverse Events

13.1 Adverse Event (AE)

Any untoward medical occurrence or effect in a patient treated on a trial protocol, which does not necessarily have a causal relationship with this treatment.

13.2 Serious Adverse Event

An adverse event that:

- Results in death
- Is life threatening
- Requires admission to hospital or prolongation of hospitalisation
- Results in persistent or significant disability/incapacity
- Is otherwise medically significant
- Return to theatre or ITU

13.3 Expected morbidity

13.3.1 Expected morbidity following PRODOSE intervention:

- No adverse events related to the intervention are expected
- Adverse event related to cardiac surgery / cardiac disease include:
  - Prolonged ICU treatment
  - Excess blood loss
  - Stroke
  - Need for permanent pace maker
  - Infection

As with all major surgery there is also a risk of death. The risk of in-hospital death with for the PRODOSE trial is expected to be 1-3%, in keeping with the institutional mortality for first time elective cardiac surgery on cardiopulmonary bypass.

No additional risks specific to the PRODOSE trial have been identified.

All in-hospital deaths will be reviewed by the Sponsor and the Trial Steering Committee.

Recommended protocol amendment:

1. Increase sample size by 28 patients overall (adjusted for drop-out rate to a sensible value). The adaptive design needs 212 in total but we add 16 more for drop-outs.

2. Modify the CRFs to properly include a more detailed record of blood loss at 4, 6, 12 hours and at drain removal or 24h, whichever comes first (crucial point is at 6 hours). Re-capture all of these blood loss measures for the 4 patients that were randomised from the electronic ICU charts.

3. Re-state that the algorithm protamine dose calculation remains unchanged and the control group dose calculation based on initial heparin dose rather than cumulative dose. The justification for this is:

    - The precision of the r-time is lower than we expected. Therefore, the variance in the initial calculation could have been underestimated slightly. In the new calculation we have used the fact that r-times are measured in minutes with 1 decimal place. The variance assumed to be 76 seconds based on the De Meesters paper would correspond with 1.26. We have rounded this value up to 1.4 to be conservative. We still aim at 90% power on the primary endpoint and the hypothesis that the algorithm reduces r-time in at least 15%.

    - The new increased sample size allows us to have 80% power to detect a 17% reduction in blood loss at 6 hours (one of our secondary endpoints). The 17% reduction is a likely value based on our pilot. Blood loss is the clinically relevant end point as reduced blood transfusion rates decrease patient risk.

    - The operating characteristics of the design are preserved. We have a 4% Type I error rate and a 4% chance of stopping the trial due to futility under the null and 90% power on the primary question. The fact that the adaptive design aims at preserving power after the interim allows for an efficiency gain of 10 less patients needed in our sample size change. If the r-time in algorithm group is in fact 20% higher than in the control group the trial will stop at the interim with 86% probability.

    - The interim analysis is now planned at patient 114. The response-adaptive change in randomisation ratio is expected to preserve power as much as possible without exposing patients to a worse arm. If the variance in r-time was still underestimated the change in the randomisation ratio will ensure that power is maximised given the size of the trial. This has the added advantage of taking care of the the possibility that variances per arm might be very different - in this case the sample size of the arm with less variance can be expected to be reduced.

East of England - Cambridge South Research Ethics Committee
The Old Chapel
Royal Standard Place
Nottingham
NG1 6FS

17 December 2018

Carol Freeman
Clinical Project Manager
Papworth Trials Unit Collaboration
Research and Development Department
Royal Papworth Hospital
Cambridge
CB23 3RE

Dear Ms Freeman,

**Study title:**	**Algorithmic Protamine Dosing for Reversal of Heparin after Cardiopulmonary Bypass (PRODOSE)**
**REC reference:**	**17/EE/0460**
**Protocol number:**	**P02337**
**Amendment number:**	**SA01**
**Amendment date:**	**23 November 2018**
**IRAS project ID:**	**231790**

The above amendment was reviewed by the Sub-Committee in correspondence.

Ethical opinion

The members of the Committee taking part in the review gave a favourable ethical opinion of the amendment on the basis described in the notice of amendment form and supporting documentation.

The members of the Sub-Committee were in agreement that the Substantial Amendment did not raise any material ethical issues.

Approved documents

The documents reviewed and approved at the meeting were:

Document	*Version*	*Date*
Notice of Substantial Amendment (non-CTIMP)	SA01	23 November 2018
Research protocol or project proposal [Clinical Trial Protocol PRODOSE]	2	07 November 2018

Membership of the Committee

The members of the Committee who took part in the review are listed on the attached sheet.

Working with NHS Care Organisations

Sponsors should ensure that they notify the R&D office for the relevant NHS care organisation of this amendment in line with the terms detailed in the categorisation email issued by the lead nation for the study.

Statement of compliance

The Committee is constituted in accordance with the Governance Arrangements for Research Ethics Committees and complies fully with the Standard Operating Procedures for Research Ethics Committees in the UK.

We are pleased to welcome researchers and R & D staff at our Research Ethics Committee members' training days – see details at http://www.hra.nhs.uk/hra-training/

**17/EE/0460:**	**Please quote this number on all correspondence**

Yours sincerely,

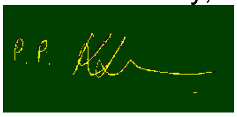

Dr Leslie Gelling
Chair

E-mail: nrescommittee.eastofengland-cambridgesouth@nhs.net

Enclosures: *List of names and professions of members who took part in the review*

Copy to: *Ms Carol Freeman, Papworth Hospital NHS Foundation Trust*
Dr Florian Falter, Papworth Hospital NHS Foundation Trust
