# Supplementary material for: Optimal protamine dosing after cardiopulmonary bypass: The PRODOSE adaptive randomised controlled trial
Source: PLoS Med. 2021 Jun 7;18(6):e1003658. doi: 10.1371/journal.pmed.1003658 (PMC8216535; doi:10.1371/journal.pmed.1003658)
Supplement: S1 Checklist — ACE, Adaptive designs CONSORT Extension; CONSORT, Consolidated Standards of Reporting Trials. (DOCX) [file pmed.1003658.s004.docx]

| **Section/Topic** | **Item no** | **Checklist item** | **Page no** |
| --- | --- | --- | --- |
| **Title and abstract** | 1a | Identification as a randomised trial in the title | Title page |
|  | 1b | Structured summary of trial design, methods, results, and conclusions (for specific guidance see ACE checklist for abstracts) | Abstract  Para 1 – 5 |
| **Introduction** |  |  |  |
| Background and objectives | 2a | Scientific background and explanation of rationale | Intro.  Para 1 – 2 |
|  | 2b | Specific objectives or hypotheses | Intro.  Para 3 |
| **Methods** |  |  |  |
| Trial design | 3a | Description of trial design (such as parallel, factorial) including allocation ratio | Methods  Para 1, 4 |
|  | 3b« ǂ | Type of adaptive design used, with details of the pre-planned trial adaptations and the statistical information informing the adaptations | Methods  Para 4, 5 |
|  | 3c«3b ‡ | Important changes to the design or methods after trial commencement (such as eligibility criteria) outside the scope of the pre-planned adaptive design features, with reasons | Methods  N/A |
| Participants | 4a | Eligibility criteria for participants | Methods  Para 2 |
|  | 4b | Settings and locations where the data were collected | Methods  Para 1 |
| Interventions | 5 | The interventions for each group with sufficient details to allow replication, including how and when they were actually administered | Methods  Para 5 – 7 |
| Outcomes | 6a ‡ | Completely define pre-specified primary and secondary outcome measures, including how and when they were assessed. Any other outcome measures used to inform pre-planned adaptations should be described with the rationale | Methods  Para 9 |
|  | 6b ‡ | Any unplanned changes to trial outcomes after the trial commenced, with reasons | Methods  N/A |
| Sample size and operating characteristics | 7a ‡ | How sample size and operating characteristics were determined | Methods Para 10 |
|  | 7b ‡‡ | Pre-planned interim decision-making criteria to guide the trial adaptation process; whether decision-making criteria were binding or non-binding; pre-planned and actual timing and frequency of interim data looks to inform trial adaptations | Methods Para 4 S2 Append |
| **Randomisation** |  |  |  |
| Sequence generation | 8a | Method used to generate the random allocation sequence | Methods Para 4 |
|  | 8b ‡ | Type of randomisation; details of any restriction (such as blocking and block size); any changes to the allocation rule after trial adaptation decisions; any pre-planned allocation rule or algorithm to update randomisation with timing and frequency of updates | Methods Para 4 |
| Allocation concealment mechanism | 9 | Mechanism used to implement the random allocation sequence (such as sequentially numbered containers), describing any steps taken to conceal the sequence until interventions were assigned | Methods Para 4 |
| Implementation | 10 | Who generated the random allocation sequence, who enrolled participants, and who assigned participants to interventions | Methods Para 4 |
| Blinding | 11a | If done, who was blinded after assignment to interventions (for example, participants, care providers, those assessing outcomes) and how | Methods Para 4 |
|  | 11b | If relevant, description of the similarity of interventions | Methods Para 5, 6 |
|  | 11c ǂ | Measures to safeguard the confidentiality of interim information and minimise potential operational bias during the trial | Methods  Para 11 |
| Statistical methods | 12a ‡ | Statistical methods used to compare groups for primary and secondary outcomes, and any other outcomes used to make pre-planned adaptations | Methods Para 12 S2 Append |
|  | 12b« ǂ | For the implemented adaptive design features, statistical methods used to estimate treatment effects for key endpoints and to make inferences | Methods Para 12 S2 Append |
|  | 12c«2b | Methods for additional analyses, such as subgroup analyses and adjusted analyses | Methods Para 12 S2 Append |
| **Results** |  |  |  |
| Participant flow (a diagram is strongly recommended) | 13a ‡ | For each group, the numbers of participants who were randomly assigned, received intended treatment, and were analysed for the primary outcome and any other outcomes used to inform pre-planned adaptations, if applicable | Results Para 2 Fig 1 |
|  | 13b | For each group, losses and exclusions after randomisation, together with reasons | Results Para 2 Fig 1 |
| Recruitment and adaptations | 14a ‡ | Dates defining the periods of recruitment and follow-up, for each group | Results Para 2 |
|  | 14b † | Why the trial ended or was stopped | Results Para 2 |
|  | 14c ǂ | Specify what trial adaptation decisions were made in light of the pre-planned decision-making criteria and observed accrued data | Results Para 1 |
| Baseline data | 15a«15 † | A table showing baseline demographic and clinical characteristics for each group | Table 1 |
|  | 15b ǂ | Summary of data to enable the assessment of similarity in the trial population between interim stages | N/A |
| Numbers analysed | 16 † | For each group, number of participants (denominator) included in each analysis and whether the analysis was by original assigned groups | Results Para 2 |
| Outcomes and estimation | 17a † | For each primary and secondary outcome, results for each group, and the estimated effect size and its precision (such as 95% confidence interval) | Results Para 3 – 5 Table 3 |
|  | 17b | For binary outcomes, presentation of both absolute and relative effect sizes is recommended | Results Para 3 – 5 Table 3 |
|  | 17c ǂ | Report interim results used to inform interim decision-making | Results Para 1 |
| Ancillary analyses | 18 | Results of any other analyses performed, including subgroup analyses and adjusted analyses, distinguishing pre-specified from exploratory | N/A |
| Harms | 19 | All important harms or unintended effects in each group (for specific guidance see CONSORT for harms) ^1^ | Results Para 8 |
| **Discussion** |  |  |  |
| Limitations | 20 † | Trial limitations, addressing sources of potential bias, imprecision, and, if relevant, multiplicity of analyses | Discussion Para 5 |
| Generalisability | 21 † | Generalisability (external validity, applicability) of the trial findings | Discussion Para 4 |
| Interpretation | 22 | Interpretation consistent with results, balancing benefits and harms, and considering other relevant evidence | Discussion Para 1, 2, 7 |
| **Other information** |  |  |  |
| Registration | 23 | Registration number and name of trial registry | Methods Para 1 |
| Protocol | 24a«24 | Where the full trial protocol can be accessed | Methods  Para 12 S4 Append |
| SAP and other relevant trial documents | 24b ǂ | Where the full statistical analysis plan and other relevant trial documents can be accessed | S2 Append |
| Funding | 25 | Sources of funding and other support (such as supply of drugs), role of funders | Funding |

SAP, statistical analysis plan; ACE, Adaptive designs CONSORT Extension;

“X« Y” means original CONSORT 2010 item Y has been renumbered to X;

“X«” means item reordering resulted in new item X replacing the number of the CONSORT 2010 item X.

ǂ New items that should only be applied in reference to ACE;

‡ Modified items that require reference to both CONSORT 2010 and ACE;

‡‡ Replacement (modified) item that only requires reference to ACE;

† Item wording remains unchanged in reference to CONSORT 2010 but we expanded the ACE explanatory text to clarify additional considerations for certain adaptive designs. These unchanged items require reference to CONSORT 2010 except item 14b.

**Citation:**

Dimairo M, Pallmann P, Wason J, Todd S, Jaki T, Julious SA, Mander AP, Weir CJ, Koenig F, Walton MK, Nicholl JP, Coates E, Biggs K, Hamasaki T, Proschan MA, Scott JA, Ando Y, Hind D, Altman DG; ACE Consensus Group. The Adaptive designs CONSORT Extension (ACE) statement: a checklist with explanation and elaboration guideline for reporting randomised trials that use an adaptive design. BMJ. 2020 Jun 17;369:m115. PMID: 32554564; PMCID: PMC7298567.

Dimairo M, Pallmann P, Wason J, Todd S, Jaki T, Julious SA, Mander AP, Weir CJ, Koenig F, Walton MK, Nicholl JP, Coates E, Biggs K, Hamasaki T, Proschan MA, Scott JA, Ando Y, Hind D, Altman DG; ACE Consensus Group. The adaptive designs CONSORT extension (ACE) statement: a checklist with explanation and elaboration guideline for reporting randomised trials that use an adaptive design. Trials. 2020 Jun 17;21(1):528. PMID: 32546273; PMCID: PMC7298968.
