# Supplementary material for: Optimal protamine dosing after cardiopulmonary bypass: The PRODOSE adaptive randomised controlled trial
Source: PLoS Med. 2021 Jun 7;18(6):e1003658. doi: 10.1371/journal.pmed.1003658 (PMC8216535; doi:10.1371/journal.pmed.1003658)
Supplement: Attachment — Submitted filename: PRODOSE_response to reviewers_R3.pdf [file pmed.1003658.s005.pdf]

21 December 2020

Dear Dr. Veitch,

RE. PMEDICINE-D-20-05286R1 Decision – [EMID:e550eba71157b99f]

On behalf of the PRODOSE authorship group, I extend our thanks to you and your expert reviewers for your insightful suggestions and commentary regarding our recently submitted manuscript, '*Optimal protamine dosing after cardiopulmonary bypass: the PRODOSE adaptive randomised controlled trial*'. Having considered and made various changes in line with your requests, we consider it much improved, and hope it now meets the justifiably high standard for publication in *PLoS Medicine*. Naturally, if it does not, we would be delighted to make further revisions as you see fit.

All changes to the manuscript have been made using tracked changes in the attached document. Additionally, I shall address specific comments here in a stepwise fashion. Our responses are formatted in *italics*.

Requests from the editors

Please structure the abstract using the *PLOS Medicine* headings (Background, Methods and Findings, Conclusions – "Methods and findings" is a single subsection). In the last sentence of the Abstract Methods and Findings section, please include a brief note about any key limitation(s) of the study's methodology.

The abstract has been structured in keeping with the PloS Medicine *style guide. Additional material regarding the limitations of the trial design has been included.*

As recommended by one reviewer, we'd recommend you update the abstract to explain a bit better how the adaptive nature of the trial design modified the design at the interim analysis (this does not need to go into substantial detail). We'd also ask that all the statistical presentations clearly indicate what the different

Postal address:
151 Barry Street, PARKVILLE VIC 3010

Email:
lachlan.miles@unimelb.edu.au

figures show (this is mainly a problem for the numbers given in square brackets, which we assumed were 95% CI but should be indicated).

Information has been included in the abstract about the alteration in the recruitment ratio at the time of interim analysis. As you suggested we have kept this brief, but we would be happy to expand upon it if you felt it necessary. We have also clarified the statistical presentation as mean [SD] and median [IQR] depending on normality.

Please reformat the citation style into PLOS Medicine's format (should be straight forward if using referencing software) – this should use callouts formatted as sequential numerals in square brackets (not superscript or round brackets).

All references and callouts have been reformatted using the PloS Medicine style as encoded into the Mendeley database.

At this stage, we ask that you include a short, non-technical Author Summary of your research to make findings accessible to a wide audience that includes both scientists and non-scientists. The Author Summary should immediately follow the Abstract in your revised manuscript. This text is subject to editorial change and should be distinct from the scientific abstract.

We have included the Author Summary following the abstract. Please feel free to make whatever editorial changes you think necessary.

We'd ask the authors to clarify whether the secondary outcomes as set out in the manuscript match up against what is set out in the trial registry record (https://clinicaltrials.gov/ct2/show/NCT03532594) which specifies that the secondary outcomes include blood loss at four hours post-surgery as measured with intercostal drain output, and blood products usage at 24 hours. If not, please make sure all analyses for prespecified secondary outcomes (as set out in the original protocol) are included in the published paper. If the trial registry record needs to be updated, that can be done retrospectively.

We can confirm that the clinically relevant, pre-defined secondary outcomes of blood loss at four hours and transfusion requirement at 24 hours that are listed in the trial registry record are the same as those reported in the manuscript. The abstract in particular has been slightly revised to reflect this more precisely. The trial registry record should not need to be updated.

Many thanks for using the ACE guideline to support trial reporting - please also append (as a supporting information file) the ACE checklist.

As requested, we have uploaded a completed ACE checklist as a supporting document.

Per the author guidelines for papers reporting results of a trial, please also upload as supporting information a copy of the original trial protocol for the PRODOSE trial.

As requested, we have uploaded the final version of the trial protocol approved by the Human Research Ethics Committee as a supporting document, together with a minor protocol amendment updating the sample size.

Reviewer #1

Patients were excluded if they were < 18 years old, had a total body weight > 120 kg (due to unpredictable heparin requirements in obese individuals), or were dialysis dependent. The reviewer feels that protamine dosing mistakes can have more serious consequences in the excluded patients. This was mentioned briefly that their findings cannot be "generalized", but they should rephrase that these patients should be more closely studied.

We acknowledge the concerns of the reviewer. We have made a small amendment to paragraph four of the discussion where we specifically acknowledge the study design and exclusion criteria when discussing the generalisability of the study. Additionally, while the study limitations paragraph already made reference to inability to generalise these results to the populations of interest, this has been amended to emphasise that these groups need to be the subject of further prospective evaluation.

The reviewer does not fully understand why the authors excluded the patients on heparin infusion before surgery. Again, the impact of protamine management is more likely to be important in complex cases.

We thank the reviewer for this most insightful question. We very much agree with their sentiment about protamine dosing management in complex cases. However, the trial was designed to prospectively evaluate the performance of the algorithm in a population at low risk of bleeding. As we note in the first paragraph of the discussion:

> *"Our exclusion criteria were chosen to minimise the risk of post-operative bleeding so that the effect of the intervention on TEG r-time could be investigated without the potential confounding effects of major haemorrhage that is inherent to certain cardiac surgical procedures and patients."*

Patients who are on a pre-operative heparin infusion are patients with an inherently higher risk of bleeding, made worse by the residual anticoagulant effects of heparin in the pre-bypass period. Additionally, if heparin

concentration is not known (i.e. the patient is on, or has recently been on an unfractionated heparin infusion), then accurate estimation of 'starting' heparin quantity for input into the model is not possible, leading to an inherent risk of protamine dosing error. We have not yet performed the necessary translational or benchtop work to ensure model accuracy in this scenario. Accordingly, it was felt unethical to include this population given our stated objectives.

Using a control post-protamine r-time of 4.2 minutes, a standard deviation of 1.27 minutes and a minimum clinically relevant effect size of 15%.

The definition of clinical relevance is unclear. Why is a 1.27-min difference clinically important? On page 30, the authors stated that FFP or PCC is given in the case of R-time above 8 min, which is far from 4.2 +/- 1.27 min.

We thank the reviewer profusely for these critical observations – this is evidence of <u>clear</u> miscommunication on our part. Meesters et al. performed their viscoelastic assay with a ROTEM (INTEM) device, as opposed to TEG (the device used for this study). As noted in the manuscript, it was on these observed control group values and observed differences that the study was powered. These devices have almost exactly the same mechanism of operation but have different reference ranges (as outlined by Nielsen in 2007 [Blood Coagul Fibrinolysis 2007;13(3):247-52] and now referenced in the manuscript). An INTEM CT value of 4.2 ± 1.27 minutes on ROTEM is converted to approximately 8 minutes ± 2.5 minutes (the upper limit of the normal reference range) when converted to TEG, hence the difference between the 'predicted' mean and the observed.

The observed standard deviation was also identified from Meesters et al. We again neglected to specify the necessary conceptual conversion in the manuscript. The 'minimal clinically relevant difference' described in the methods is also ambiguous. It is more correct to state this was the 'predicted' difference based on our interpretation of Meesters et al., rather than what we considered to be clinically relevant. We apologise for these problematic descriptions and have clarified them in the manuscript.

Reviewer #2

As the relative difference between arms was greater than 7.5% (21.1%), we adapted the randomisation ratio for the remaining participants to be 1:1.33 as per the design

Were the authors aware that the intervention groups had a greater than 7.5%, suggesting a superiority at the preliminary evaluation point? It was unclear how the authors and statisticians decided to make a 1:1.33

ratio. Please revise the sentences, so that readers can clearly understand the sample size changes, and their justifications.

We thank the reviewer for highlighting the lack of clarity on this point in the manuscript, part of it caused by design considerations being presented in an appendix to the original submission as opposed to in the main text. As mentioned in our supplemental methods, the randomisation ratio of 1:1.33 was triggered in our design in the case of our original assumptions being violated and it was the maximum ratio that maintained study power (at 90%) under assumptions of an exponential distribution whilst being still being consistent with the ethical deviation of the ratio observed in the original design when assumptions hold. The 1:1.33 ratio was in the 85% percentile of selected randomisation ratios under the alternative hypothesis of a treatment effect using standard assumptions by Zhang and Rosenberger (2006, Reference 15 in the manuscript). This was considered to be a suitable deviation in both an ethical and statistical sense at the time of designing the study in case our original assumptions were to fail, as they did, to better capture the original design's properties. To aid in clarity we have moved this justification into the main text as suggested also by Reviewer #2.

Also, note that only the trial statisticians were aware of this difference at the interim time, the trial did not include an efficacy stopping pre-specified rule but instead a randomisation ratio update to favour the superior trial if the trial was to continue to its final stage with an observed superiority of a treatment arm.

Despite this reduction in protamine dose, we found no evidence of clinically or statistically significant difference in post-operative bleeding or transfusion requirement.

The authors chose relatively straight forward cases, and thus the overall impact on clinical endpoints might have been minimal. Secondly, the control group received a 100-mg more protamine than in the intervention group. This 'excess' dose may not have had a prolonged impact on clinical hemostasis because protamine disappears rapidly (<5 min) from circulation (Butterworth J, et al. Ann Thorac Surg 2002). The authors obtained the TEG samples in 3 min after protamine administration, which demonstrated a transient negative impact on the TEG R-time. This possibility should be mentioned in the discussion.

The reviewer is indeed correct regarding the biological half-life of protamine, particularly its rapid clearance from the circulation as outlined by Butterworth et al. However, despite the short biological half-life of protamine, it is hypothesised that the clinical anticoagulant effects of protamine persist once it has left the circulation. An analysis of 16 studies performed by Boer et al. (Br J Anaes 2018;120(5):914-27) shows a clear and dose-dependent linear relationship between protamine-to-heparin ratio and 12-hour blood loss post-operatively, a signal replicated in our own retrospective work (Kunz et al., Perfusion 2018;33:445-52). While the reasons for this are somewhat unclear, recent prospective work by Olsson et al. (Scand Cardiovasc J,

2019;6:355-60) suggests platelet dysfunction persists for at least 20 minutes after protamine dosing, with recovery seen by time of arrival in ICU. The net summation of these observations is that the clinical effects of protamine excess persist beyond the biological half-life of the drug, and that protamine excess is generally recognised as a contributor to postoperative bleeding. A summation of the reviewers point together with a brief discussion of this counterargument is included in the study limitations.

Table 3: What were the duration of observation for 'Postoperative mediastinal drainage' and 'Postoperative RBC requirement'?

As mentioned in the manuscript, postoperative mediastinal drainage was measured at 4 hours, and postoperative RBC requirement was measured at 24 hours. The table caption has been updated to reflect this.

P32, At this point the effect size was assessed having noted a greater than 7.5% difference in treatment arms, the second 114 group assignments was sampled at a ratio of 1:1.33. If a difference of this magnitude had not been noted, the ratio would have remained at 1:1. Please explain why a greater than 7.5% was used as a cut-off to increase the sample size of the intervention when it already appeared to be superior? What would have happened if this was not changed?

As mentioned in the supplemental methods our design chose to use a 7.5% threshold as it the value that made the design in case of non-normality closer to our design when normality assumptions hold, specifically in terms of the allocation ratios and power. In simulations during the design stage, it was noted that in case of deviations from normality the randomisation change in our original design was almost never triggered even if the presence of an observed superiority of the treatment arm. To better include the case of deviations from normality into the design, the randomisation ratio was allowed to be adapted in that case if the observed treatment difference at the interim was half the minimum relative clinically significant difference (7.5%) the randomisation ratio would then be set to 1:1.33 in favour of the beneficial treatment arm. If the difference were less than 7.5% the randomisation ratio would be maintained at 1:1. This was basically following what the design would have done in case of normality in the data and superiority of one of the arms. Significant deviations from normality were determined through a significant ($p<0.05$) Shapiro-Wilk Test. This threshold was found in simulations to ensure a study power of 90% under assumptions of an exponential distribution. To minimise bias, only the primary endpoint and safety variables were analysed at the interim. The interim and final analysis were performed by two different statisticians. If the primary outcome did not exceed the limits for safety or futility, the randomisation ratio would be adapted to increase recruitment to the superior arm based on interim data, while preserving study power (S2 Appendix). The clinical investigators remained blinded to any change of randomisation strategy until the end of the study.

Reviewer #2

Abstract

I think that a bit more explanation on what is meant by "adaptive" would be good. Perhaps "two-stage with revised randomisation ratio" or something along these lines.

What do you report in the "[]". Are these 95% CIs, or SDs, or SEs? This comment is also valid for the main text.

We have amended the abstract to better describe the design of the study, the values reported in [] are IQR, and those in () are SD or proportion. These have been reported alongside measures of central tendancy throughout the manuscript to ensure sufficient clarity.

Main text

Trial design: "An interim analysis was scheduled to check for safety, consider a pre-defined futility rule and to adapt the randomisation ratio based on the primary outcome data at that point". I agree that all details are in Appendix 2. However, some information needs to come in the main text, including the potentially revised randomisation ratio, perhaps the thresholds for futility etc

We thank the reviewer for highlighting a potential lack of detail in the methods. We have added additional information to the methods that was included in the appendix. We have focused on including any relevant information regarding the reasoning and justification of the altered randomisation ratio and relevant interim stopping criteria and threshold. We hope this is now to your satisfaction.

Statistical analysis

"Primary efficacy analysis was carried with a re-randomisation-based method, ensuring type I error was preserved despite deviations in assumptions and taking the adaptive design into account." Please could you clarify why you used this re-randomisation method? Was it not possible to use the Mann-Whitney test (or something equivalent), perhaps even after transformation of the primary endpoint if not normally distributed?

The re-randomisation test was chosen as it allowed for us to adjust for the observed violations in assumptions from our data and the study design in a single method. It is also known to be robust (under general conditions) when changing a randomisation ratio during a trial, so we felt it was reassuring result to have significance with this test in any case (please see the paper by Simon & Simon 2011 PMID – 21769160, for further discussion of the topic). Non-parametric tests in isolation may not have fully considered design aspects such as changes in randomisation ratio which may introduce bias. We also did attempt to transform our data but

there was not a straightforward transformation that led to favourable characteristics. To better highlight this reasoning, we have included more detailed explanation in the methods.

Results

"We found that the distribution of the primary outcome deviated significantly from normality (intervention, p = 0.0061; control, p = 0.011)". What are these p-values? Test for normality? If yes which test?

"As the relative difference between arms was greater than 7.5% (21.1%)" So what is 21.1%, the observed relative difference at that point?

We thank the reviewer for highlighting the lack of clarity in this text. The p-values represent the results from the test of normality (Shapiro-Wilk) and 21.1% was indeed the relative difference at that point. We have amended the sentences to better reflect what the values represent and how they were calculated.

Reviewer #3

Abstract

Methods, please include how many patients were in the treatment/model group and how many in the fixed ratio group.

This information has been added in response to the reviewer's request.

Results, the authors should clarify the type of transfusion and units.

This information has been added in response to the reviewer's request. The a priori *declared outcome was transfusion requirement as a binary measure, rather than number of units administered.*

Conclusion, a comment/conclusion should be added that there was no difference between groups in blood loss and blood transfusion.

This information has been added in response to the reviewer's request.

The recent EACTS/EACTA/EBCP guidelines (Wahba et al. 2020) recommend individualised heparin and protamine management in order to reduce postoperative coagulation abnormalities and bleeding complications in cardiac surgery with CPB. This should be added to the second paragraph, particularly as it deviates from a fixed (1:1) dosing recommendation from earlier guidelines (Pagano et al. 2016).

We thank the reviewer for drawing this to our attention. However, our interpretation of the guidance differs. While the guidelines do make reference to the existence of individualised strategies as the reviewer rightly points out, the end conclusion made in the relevant section (p. 226) reads:

> *"In summary, protamine should be given in a ratio of 0.8 – 1.0 of the initial doses of heparin".*

Additionally, the Class IIA, Level B recommendations regarding protamine administration (p. 227) of the guideline state only that:

> *"Protamine overdosing should be avoided to reduce postoperative coagulation abnormalities and bleeding in cardiac surgery with CPB".*

While we have updated the Pagano reference to reflect the updated guideline by Whaba et al., and thank the reviewer very much for their insights, we respectfully decline to state that the EACTS/EACTA/EBCP explicitly recommend the use of individualised protamine dosing strategies following CPB.

The Kjellberg study, introducing an algorithm to calculate protamine in a randomised controlled trial, should be listed in the second paragraph of the introduction (reference 31). The results of this RCT revealed that there was a reduced dose of protamine, but blood loss and transfusion rates were similar in the intervention group when compared to the control group.

A reference to Kjellberg et al. has been added to the second paragraph of the introduction at the request of the reviewer.

Please clarify the type of heparin used and the manufacturer. Different manufacturers may produce heparins with different coagulation effects.

We have included this information at the request of the reviewer. Different heparin manufacturers were used at each of the two sites. However, we do not view it as a study limitation. Heparin was dosed using international units, which are a measure of heparin activity (specifically the equivalent of 0.002 mg of pure heparin), rather than absolute molecular weight of heparin, which varies considerably due to heterogenous polysaccharide chain length. Accordingly, we are confident that there is no difference in the amount of anticoagulant 'activity' administered to patients randomised at the different sites, and even if there were, the randomisation process should have addressed this source of bias.

What technique of residual blood management was used at the end of CPB: direct re-transfusion of unprocessed blood, re-transfusion of processed blood or both? This may have an effect on coagulation factors administered to patients at the end of CPB and should therefore be clarified, particularly if there were difference between groups.

As cell salvage is not routinely used at either institution for low-risk cases, participants received re-transfusion of unprocessed blood. The protocol stipulated that heparin was only administered for unprocessed pump blood. As the reviewer requests, this has been clarified in the manuscript. However, a prohibition on cell salvage was not made in the protocol, and those patients who had a return to theatre for bleeding would likely have been reinfused processed blood. We did not collect prospectively any information on the use of cell salvage in the small numbers of patients that required a return to theatre.

Please include actual perioperative results of aPTT, PT, fibrinogen, ATIII levels, kaolin and heparinase TEGs and platelet counts in the results section, in addition to the presented p-values.

The aPTT, PT, fibrinogen and platelet count results are now listed in the results section at the request of the reviewer. The TEG results are included as table 4, together with a brief discussion in the results section. ATIII was not collected as part of the study protocol.

The number of FFPs and platelet concentrates administered in patients of each group should be added.

We thank the reviewer for this suggestion, but with the greatest respect, submit that it is not necessary, and would present an unnecessary distraction for the reader. As noted from the existing results, very few patients in either group received FFP or platelets (platelets (5 [4.2%] vs. 2 [1.9%]; p = 0.451, cryoprecipitate/fibrinogen concentrate (0 [0%] vs. 3 [2.8%]; p = 0.103). In light of such a small requirement the numbers of units administered would not yield any meaningful clinical or statistical significance and would be substantially confounded by those patients that required a return to theatre for surgical bleeding. Nevertheless, if the reviewer or you felt that these data were essential for the publication to meet an acceptable standard, we would be delighted to reconsider.

Was there any difference between PRC transfusions between groups beyond 24hrs?

These data were not collected. The study protocol stipulated that follow-up would end at 24-hours postoperatively. There were sound methodological reasons for this, as noted on page 9 of the manuscript where we state:

> *"Timepoints for post-operative measurement of mediastinal/pleural drainage, transfusion requirement and return to theatre were based on previous studies. Observed outcomes beyond these times were unlikely to be primarily related to heparin/protamine interactions at the time of surgery."*

Appropriate references are provided justifying this approach. While we could return to the medical record to extract these data, it would be an exploratory post hoc *analysis that would be confounded by factors other than the intervention. Accordingly, we would prefer not to include these data.*

In order to address reversal of heparin it would also be important to measure serum concentrations of heparin levels after the protamine administration. This limitation should be discussed.

The reviewer is absolutely correct in their statement that it is important to address correction of heparin as part of the study protocol. However, we submit that we have done so by the integration of heparinase TEG as part of our experiment. Were we not to have included heparinase TEG as a metric in this study, we agree that failure to measure heparin concentration would be a major limitation. However, during the study design we made a conscious choice to preference this metric over direct measurement of heparin concentration due to the similarity to the primary outcome. As we note on page 11 and 12 of the manuscript:

> *"No difference in the ratio of post-CPB heparinase TEG to kaolin TEG ratio (p = 0.747) was seen in the intervention group, relative to the control group."*

As there was no difference in this ratio, we can directly infer that any difference between the groups was not due to confounding by inadequate heparin reversal in the intervention (i.e. lower dose) group, and that therefore a formal measure of heparin concentration was not required to prove our hypothesis. Again, should the reviewer or you insist that a discussion of this limitation is essential for publication, we would be delighted to reconsider.

How does the protamine dose algorithm compare with individualised point-of-care devices (e.g. Hepcon) for protamine dosing? What are potential advantages of the Hepcon method of titrating heparin and protamine? Should Hepcon guided coagulation management in cardiac surgery be compared with a protamine dose algorithm in the future?

*While there is a brief discussion of heparin titration devices in the discussion, their performance relative to our model is not the focus of this study, and we would be uncomfortable including it as a key part of our discussion. Use of the HepCon in the UK and Australia is exceedingly limited (we believe there to be only one such device in current clinical use in Australia), and therefore our trial was designed as a pragmatic assessment of our model against the perceived standard of care. While there have been some benchtop/*in-vitro *studies of how algorithm-based dosing compares to the HepCon, we believe that such a discussion would be better discussed in an accompanying editorial, if at all. At this stage, we believe that future studies comparing our model to the HepCon are premature, and the* clinical *superiority of our approach over the current standard of care should be proven before further comparisons are contemplated.*

It should be discussed if there is a potential clinical benefit for patients if protamine dose algorithms are used?

This is an interesting question posed by the reviewer, and again, one probably better suited to an editorial. At this stage, there does not appear to be a clinical benefit in using our model in the population studied, although we have discussed the limitations of our study design in ascertaining this. At present, the chief benefit that can safely be determined from our data is largely pharmacoeconomic. As we note in our manuscript, testing the algorithm in higher risk populations may yield a clinical benefit, but our present data does not allow us to make such a rhetorical leap; given what we are comfortable stating from the data we have presented, the only firm conclusion that we can discuss is that further prospective work is required.

Once again, and on behalf of the authors, we thank you and your reviewers for your most insightful observations and suggestions. On the rare points where we have respectfully disagreed, we hope that you find our reasoning acceptable. Nevertheless, and as we have stated above, should you or your reviewers consider the rebuffed changes to be essential for publication, we are delighted to make further revisions.

We hope that our changes have improved the quality of our manuscript sufficient to satisfy your concerns, and we look forward to your further correspondence.

Yours sincerely,

Dr Lachlan F. Miles
Honorary Senior Fellow
Centre for Integrated Critical Care
