# Supplementary material for: Optimal protamine dosing after cardiopulmonary bypass: The PRODOSE adaptive randomised controlled trial
Source: PLoS Med. 2021 Jun 7;18(6):e1003658. doi: 10.1371/journal.pmed.1003658 (PMC8216535; doi:10.1371/journal.pmed.1003658)
Supplement: Attachment — Submitted filename: PRODOSE_response to reviewers_Final.docx [file pmed.1003658.s006.docx]

| Postal address:  151 Barry Street, PARKVILLE VIC 3010 | Email:  lachlan.miles@unimelb.edu.au |
| --- | --- |

9 May 2021

Dear Dr. Turner,

**RE. PMEDICINE-D-20-05286R1 Decision – [EMID:e550eba71157b99f]**

On behalf of the PRODOSE authorship group, I extend our thanks to you and your expert reviewers for your insightful suggestions and commentary regarding our recently submitted manuscript, ‘*Optimal protamine dosing after cardiopulmonary bypass: the PRODOSE adaptive randomised controlled trial’*. Having considered and made various changes in line with your requests, we consider it much improved, and hope it now meets the justifiably high standard for publication in *PLoS Medicine*. Naturally, if it does not, we would be delighted to make further revisions as you see fit.

All changes to the manuscript have been made using tracked changes in the attached document. Additionally, I shall address specific comments here in a stepwise fashion. Our responses are formatted in *italics*.

**Requests from the editors**

Please adapt the wording of the data statement to "... cannot be made publicly available ..." or similar (assuming this is the case).

*We have slightly reworded the Data Sharing Agreement to conform with the suggested language.*

Please quote the study dates in your abstract.

*The requested information has been included.*

Please specify the study's primary endpoint in your abstract, we suggest around line 15. We suggest also adding a subsequent "Secondary endpoints included ..." sentence, quoting the secondary endpoints on which you report findings in the abstract.

*The requested information has been included.*

Please adapt the text around line 22 of the abstract to indicate that this is the primary study finding.

*The abstract has been ordered to place the primary outcome first in reported results. As protamine dose was not a designated primary or secondary endpoint, it has been moved to the end of the secondary endpoint section.*

Regarding the abstract and main text, we are used to seeing an effect size with associated 95% CI quoted for the primary endpoint of a trial. Is this something you can provide?

*We have now reported standard estimates of the treatment effect and associated 95% confidence interval. Given the negligible difference between our adjusted p-value (0.0016), which considers the minor change in randomisation ratio of the adaptive design, and an unadjusted p-value (0.0011), we expect this standard CI to have the expected coverage*

Please quote the trial registration number in your abstract.

*The requested information has been included.*

Please adapt the format of your author summary so that the 3 subsections each consist of about 3 bulleted points (each of 1-2 short sentences). You may find it helpful to consult one or two recent research papers in PLOS Medicine to get a sense of the preferred style.

*The author summary has been better adapted according to the preferred style. As you have suggested, each subsection now consists of three separate points, using the active voice.*

Please rename the attached CONSORT checklist "S4_CONSORT_Checklist" or similar and refer to it by this label in the Methods section (we suggest using the acronym "CONSORT" in the text, incidentally).

Please adapt the checklist so that individual items are referred to by section (e.g., "Methods") and paragraph number. Please do not use page or line numbers, as these will change in the published paper.

*We have labelled the CONSORT ACE checklist as S3 and the Trial Protocol as S4 in the Methods section. The CONSORT ACE checklist has been revised and attached.*

The first paragraph of the discussion should summarize the study. Therefore, you may wish to truncate this paragraph immediately prior to "However, it must be acknowledged ..." and the subsequent sentences could be moved to other paragraphs in which related literature and limitations are discussed.

*The requested action has been performed.*

The first paragraph of the discussion should summarize the study. Therefore, you may wish to truncate this paragraph immediately prior to "However, it must be acknowledged ..." and the subsequent sentences could be moved to other paragraphs in which related literature and limitations are discussed.

*The paragraph has been truncated as instructed. The section discussing clinical endpoints and trial design has been moved to a more appropriate pace in the discussion.*

Please revisit the discussion of blinding throughout the text, to ensure consistency. It seems that the participants were blinded, and so it may help to add a few words to explain the statement "effectively unblinded" (Discussion section).

*Descriptions of blinding (or lack thereof) have been revisited throughout the text and are now consistent, referring to the fact that “anaesthetist and surgeon were not blinded to the intervention”.*

Please remove the information on data availability, funding, and competing interests from the end of the main text. This will appear in the article metadata, via entries in the submission form.

*The relevant information has been removed.*

Please remove "table of contents" and the PubMed details from reference 41.

*The relevant reference has been revised.*

Once again, and on behalf of the authors, we thank you for your most insightful observations and suggestions. We hope that our changes have improved the quality of our manuscript sufficient to satisfy your concerns, and we look forward to your further correspondence.

Yours sincerely,

Dr Lachlan F. Miles

Honorary Senior Fellow

Centre for Integrated Critical Care
